# Machine-Learned Potentials by Active Learning from Organic Crystal Structure Prediction Landscapes

**DOI:** 10.1021/acs.jpca.3c07129

**Published:** 2024-01-26

**Authors:** Patrick W. V. Butler, Roohollah Hafizi, Graeme M. Day

**Affiliations:** School of Chemistry, University of Southampton, Southampton SO17 1BJ, U.K.

## Abstract

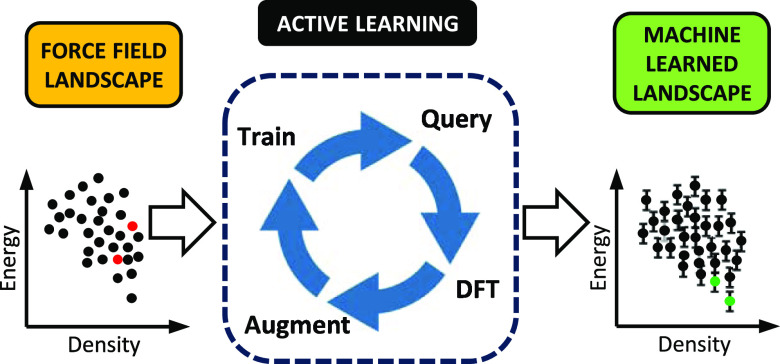

A primary challenge in organic molecular crystal structure prediction (CSP) is accurately ranking the energies of potential structures. While high-level solid-state density functional theory (DFT) methods allow for mostly reliable discrimination of the low-energy structures, their high computational cost is problematic because of the need to evaluate tens to hundreds of thousands of trial crystal structures to fully explore typical crystal energy landscapes. Consequently, lower-cost but less accurate empirical force fields are often used, sometimes as the first stage of a hierarchical scheme involving multiple stages of increasingly accurate energy calculations. Machine-learned interatomic potentials (MLIPs), trained to reproduce the results of ab initio methods with computational costs close to those of force fields, can improve the efficiency of the CSP by reducing or eliminating the need for costly DFT calculations. Here, we investigate active learning methods for training MLIPs with CSP datasets. The combination of active learning with the well-developed sampling methods from CSP yields potentials in a highly automated workflow that are relevant over a wide range of the crystal packing space. To demonstrate these potentials, we illustrate efficiently reranking large, diverse crystal structure landscapes to near-DFT accuracy from force field-based CSP, improving the reliability of the final energy ranking. Furthermore, we demonstrate how these potentials can be extended to more accurately model structures far from lattice energy minima through additional on-the-fly training within Monte Carlo simulations.

## Introduction

Molecular crystals are prevalent across a diverse range of materials applications, including optoelectronics, pharmaceuticals, and energetic materials.^1−3^ The desirable properties of these materials are often strongly tied to the crystal structure—the arrangement of the molecules in the crystal lattice—and changes solely in the crystal structure can greatly affect a wide range of the physiochemical properties of the crystals. This is seen clearly in polymorphs, which are crystals of the same compound but with different crystal structures.^4^ The properties of polymorphs often differ substantially, such that the unexpected appearance of a polymorph can result in a loss of control over material properties; the example of polymorphism in the drug Ritonavir illustrates the impact that this can have in pharmaceutical materials.^5^ Polymorphs also offer an opportunity since they allow materials to be potentially tuned to achieve enhanced properties.^6^ Thus, predicting the crystal structure of molecular crystals has become a highly coveted goal, and crystal structure prediction (CSP) is one of the primary challenges in material science and computational chemistry.

CSP methods can conceptually be divided into two parts: first, the high-dimensional lattice energy space is comprehensively sampled to identify all relevant low-energy, stable structures; and thereafter, the structures are ranked in terms of how likely they are to be observed. In general, the crystal structures are ranked based on thermodynamic stability. The resulting predicted landscapes typically contain hundreds to thousands of unique structures. As evidenced by the CSP blind tests, the best sampling methods have achieved maturity and reliably locate matches to experimental structures for rigid and moderately flexible molecules.^7−10^ By contrast, determining the relative energy ranking of predicted organic crystal structures remains a notable challenge, with often hundreds of distinct structures being within the typical energy range of polymorphism (∼7–8 kJ mol^–1^)^11^ above the global energy minimum. Differentiating these structures relies predominately on accurately evaluating the subtle balance of weak intermolecular interactions that hold organic crystals together. Additionally, thermal and entropic effects can be important for polymorphs close in energy. High-level ab initio calculations provide a measure of consistency in identifying the balance of intermolecular forces.^12−18^ However, these calculations have considerable computational cost and therefore are typically only applied to a subset of the predicted landscape or are restricted to researchers with access to very large-scale computing resources.

The large cost of density functional theory (DFT) calculations has resulted in pairwise atom–atom force fields with simple functional forms and multipole electrostatics remaining a fundamental part of CSP methods.^19^ Indeed, because of the high number of crystal structures that must be evaluated, the initial energy surface that is sampled during the first stage of organic CSP is inevitably a force field energy surface. The effectiveness of CSP in finding good geometric matches to experimental structures thus reflects the generally good structures generated by these force fields. The relative energies calculated using the force fields applied during structure generation are equally important and, if not the final energies themselves, are the basis for selecting structures for further, higher-level calculations. Therefore, it is desirable that these computationally cheaper methods be as accurate as possible. However, fitting force fields that have high accuracy across diverse structures is challenging due to their simple functional form, and compromises are often required.

A promising pathway to achieving the required high level of accuracy of organic CSP at low cost is through the use of machine-learned interatomic potentials (MLIPs), which, following training on ab initio reference data, can estimate energies and forces with the same level of accuracy but at a fraction of the cost.^20−24^ Recent developments in both theory and computing hardware have led to MLIPs being widely adopted across materials modeling, including in CSP.^25−29^ Their adoption for organic CSP, however, has been slower than in other areas due to the unique challenges of these materials. Moreover, modern MLIPs in general rely on the axiom that total energy can be decomposed into a sum of atomic energies, which are predicted based on local atomic environment descriptors. This particularly works well when the interaction is well-screened beyond the cutoff radius or when the bonding is homogeneous, as in inorganic materials. However, it cannot accurately capture interactions that occur on different scales, including the diversity of intermolecular interactions in organic crystals. Hence, while modern MLIPs excel at describing short-range interactions, they often completely neglect the longer-range interactions, including long-range dispersion and electrostatics, that extend beyond typical, computationally feasible cutoffs for the local atomic environment. Overcoming this limitation in order to capture all the relevant interactions in organic crystals requires either a more complex MLIP method^30−32^ or alternatively, the incorporation of a physical baseline that incorporates the missing long-range interactions.

A common approach to including a physical baseline is with Δ-learning, which, rather than learning total energies, focuses on learning the difference between a lower-level method, such as a force field or density functional-based tight binding (DFTB), and the higher-level method, such as DFT. With the appropriate choice of the baseline, Δ-learning combines the accurate description of long-range effects with the high-level accuracy of MLIPs for short-range interactions, which can increase the accuracy of the final model with less data.^33^ The validity of this approach has been demonstrated for organic crystals, with further extensions including multimer corrections and training separate models for the intramolecular and intermolecular components.^34−37^

Accurate MLIPs are also highly dependent on the training data collected. This is because the nonphysical functional form of MLIPs means they are typically only accurate interpolating within regions of the energy surface covered by the training data. Consequently, generating comprehensive, representative, and diverse datasets is a nontrivial problem and a key concern for MLIP development. On the one hand, large datasets allow MLIPs to cover a wide region of the energy landscape with high accuracy. On the other hand, large datasets are unfavorable because large numbers of expensive ab initio calculations are required and the computational cost of MLIP training increases. Furthermore, large randomly sampled datasets might introduce biases toward common configurations. A common approach to avoiding these pitfalls is to apply an active learning approach, where structures are iteratively added to the training set from a large pool of candidates based on the model’s predictions, with the model being retrained after each iteration to update the predictions.^25,38−48^ There are various implementations of active learning. However, for MLIPs, active learning often involves estimating the uncertainty of the predictions and adding structures with high uncertainty to the training set. Previous studies have found that active learning can significantly reduce the training set size required to achieve a certain level of accuracy of MLIP, reducing computational costs proportionally.^38^

Because active learning cannot increase the scope of the MLIP beyond that covered by the set of candidates, generating robust candidate datasets is still important. Molecular dynamics is one common method for generating these datasets. However, in the context of MLIPs for solid-state systems, CSP landscapes can provide more diverse candidates, covering a wide scope of the potential energy surface (PES) and being largely free of selection biases. As well as improving the efficiency of future CSP studies, MLIP training to CSP landscapes can leverage the excellent resource of already published CSP landscapes for those interested in developing MLIPs for crystal structure modeling.

**Figure 1:**
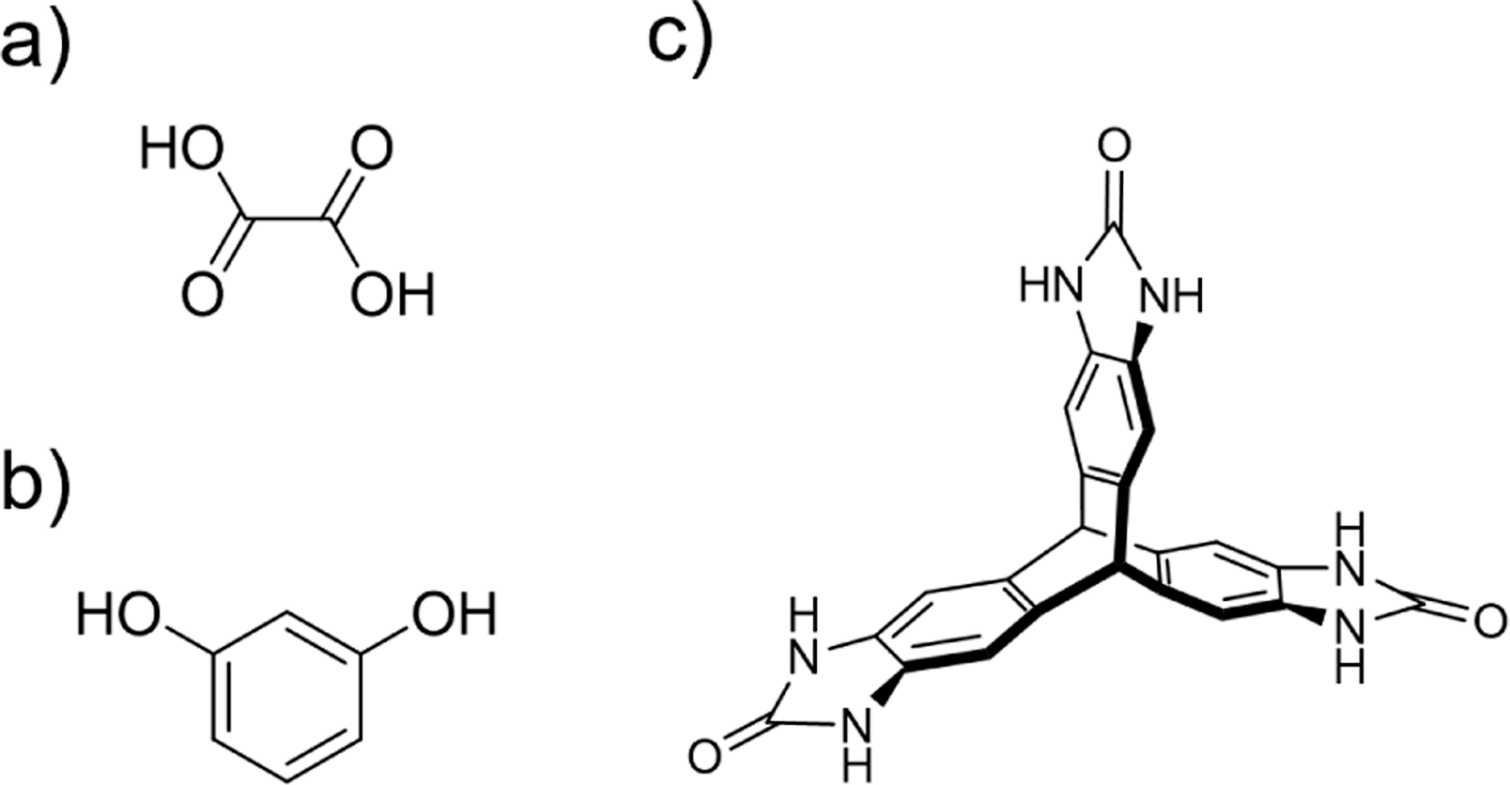
Compounds considered in this study: oxalic acid (a), resorcinol (b), and TTBI (c).

In this contribution, we investigate how best to develop MLIPs, specifically neural network potentials (NNPs), from organic CSP landscapes. We begin by examining active learning on a CSP landscape of oxalic acid (Figure 1a), investigating the effects of hyperparameters and strategies on the size and quality of the selected training set. From this, we identify an efficient approach combining active learning with Δ-learning. Thereafter, we demonstrate this approach through correcting the CSP landscapes of resorcinol (Figure 1b) and triptycene-tris(benzimidazolone) (TTBI, Figure 1c), each containing thousands of structures, to the DFT level. Finally, we detail how the potentials can be extended to describe structures far from the CSP minima by combining on-the-fly training with Monte Carlo (MC) simulations. The scheme presented here provides access to MLIPs relevant over a wide scope of the crystal packing space and with the exacting accuracy required for organic CSP in a simple, efficient, and highly automated workflow.

## Methods

**Figure 2:**
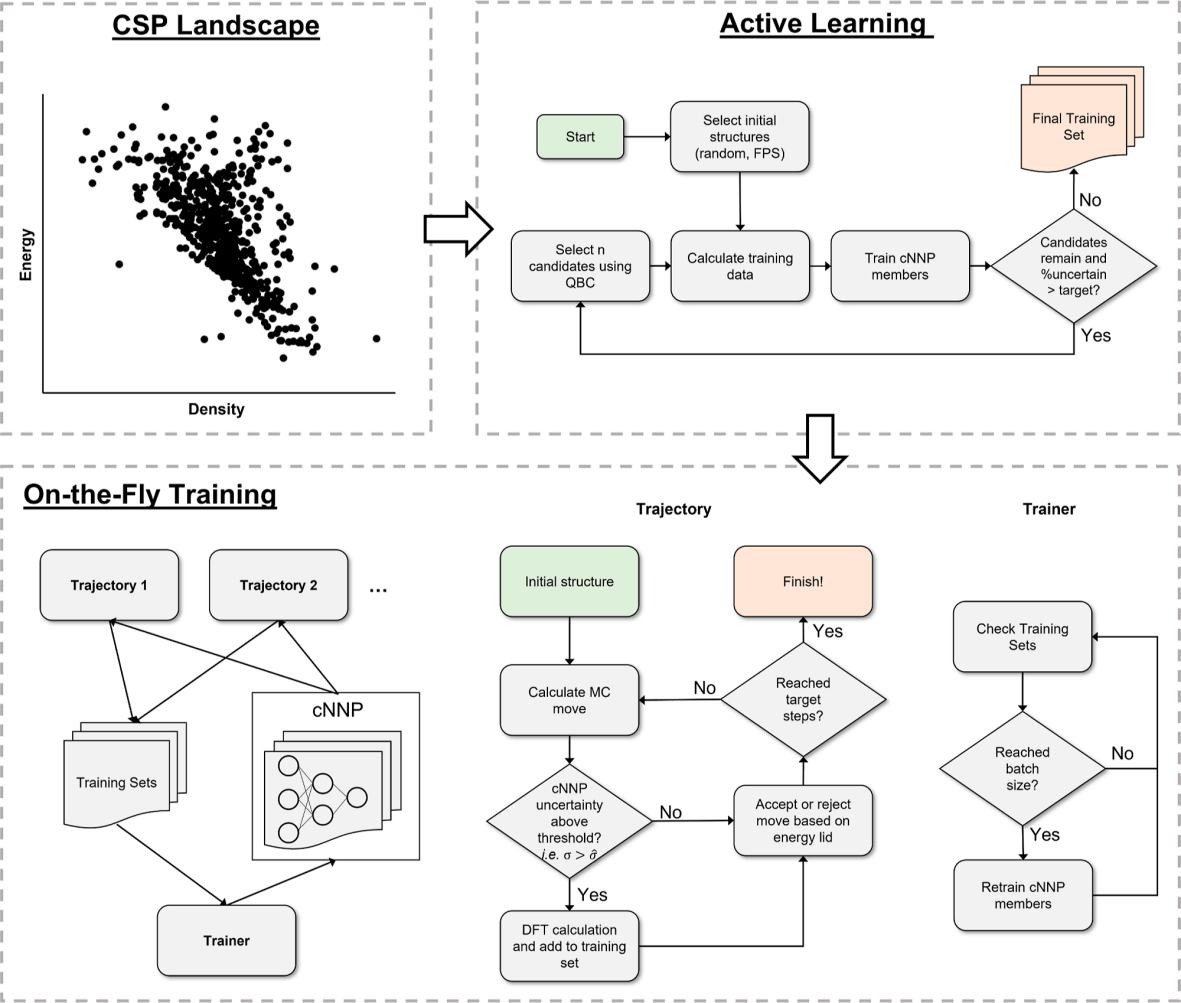
Overview of the workflow detailed. Starting from an initial CSP landscape, the active learning flowchart describes how the final training set is produced. Committee NNPs trained on this training set can then be extended through on-the-fly training. Overview of the on-the-fly training and flowcharts for the trajectory and trainer subroutines are shown. Each subsection is automated and, thus, from CSP to on-the-fly training can be achieved with minimal intervention.

An overview of the workflow described here is listed in Figure 2. The fundamental idea consists of NNPs trained by active learning using query-by-committee (QBC) techniques to identify high-uncertainty structures in CSP landscapes and MC trajectories. The CSP landscapes can be explicitly calculated for this purpose or reused from prior studies. In this work, only the oxalic acid landscape was calculated for the purpose of training NNPs. The more computationally demanding landscapes for resorcinol and TTBI were taken from earlier works.^49,50^ All landscapes were originally generated by a quasi-random sampling of the crystal packing space using our Global Lattice Energy Explorer (GLEE) code.^51^ The initial trial structures were generated from rigid molecules, and lattice energy minimized using an empirically parametrized *exp-6* potential consisting of the FIT^52−54^ parameters for describing intermolecular exchange–repulsion and dispersion combined with atom-centered multipoles obtained from a distributed multipole analysis^55^ (DMA) of the DFT-calculated molecular electron density (FIT + DMA). In the case of resorcinol, to account for the conformational flexibility, crystal structures were generated using a pool of rigid conformations and, following rigid-molecule lattice energy minimization, were fully relaxed at the dispersion-corrected DFTB level (DFTB-D3). Further details are provided in the Supporting Information.

Reference energies and forces were calculated with DFT by using the PBE exchange correlation functional with the D3(BJ) dispersion correction (PBE-D3). This method is widely used as a first DFT approximation for molecular crystal lattice energies.^10^ The calculated reference data was used to train NNPs of the Behler–Parinello high-dimensional NNP form,^56^ as implemented in the n2p2 code.^57,58^ The input to the NNPs is vectors of radial and angular atom-centered symmetry functions (ACSFs). ACSFs were selected from a larger set by a CUR decomposition following the procedure detailed by Imbalzano et al.,^59^ which offers an improvement over a general set of symmetry functions (Table S1). For oxalic acid, a total of 64 radial and angular symmetry functions per element were selected, while for TTBI and resorcinol, 128 total symmetry functions per element were selected. In all cases, a maximum radial cutoff of 8.0 Å was used. Further details of the reference calculations and NNP models are provided in the Supporting Information.

### Query-by-Committee

As pure mathematical functions, neural networks do not natively estimate uncertainties in their predictions, and thus various methods have been developed to provide these. A common approach with NNPs, and the one we use here, is to create committee NNPs (cNNPs) and obtain uncertainties via QBC. This involves training an ensemble of *n* individual models, the committee, using the same dataset but with random variations in the weight initialization of each member and/or splitting of training sets. Predictions are then made by averaging over the predictions of the individual members; for example, energies are estimated as
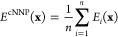
1where **x** is the descriptor vector for a given structure and *E*_*i*_ is the energy predicted by member *i* of a committee of *n* members. The uncertainty is derived from the standard deviation (σ) of the prediction between committee members. High standard deviations imply high errors for one or more of the members, indicating that the model is extrapolating beyond the training data and so reflecting the random variation between committee members. In addition to the uncertainty measure, by averaging over predictions, cNNPs have also been shown to have higher accuracy compared to a single NNP.^47^ The caveat of cNNPs is an increased cost in training and predictions. This increase can be minimized through parallelization and by storing ACSF vectors.

### Active Learning from CSP Landscapes

Candidates indicated to have high uncertainty by QBC suggest regions of the configurational space that have not been learned sufficiently accurately, given the current training set. Therefore, applying this to CSP landscapes, we iteratively add predicted structures with high uncertainty to the training set. For the initial iteration, before the cNNP is first trained, we sample the candidates either randomly or by farthest point sampling (FPS), wherein additional structures are selected based on the maximum distance in the descriptor space from the previously selected structures (the first structure is selected randomly). While ideally the model would be retrained after each new structure is added to the training set, this has an impractically high computational cost in most cases, and thus, we define a batch size for how often the cNNP is retrained, i.e., the number of structures added at each iteration of active learning.

Another important parameter is the threshold for defining high uncertainty, , which determines the structures that are added to the training set. Setting this parameter depends on the desired accuracy of the final model but is typically complicated by not knowing the relationship between the variance in the cNNP predictions and the true error a priori. Here, we define the uncertainty directly as the standard deviation of the cNNP prediction and set an uncertainty threshold in terms of the target energy units, kJ mol^–1^ per molecule (abbreviated as kJ mol^–1^ hereafter). While the uncertainties are uncalibrated, we find that a threshold of 1–2 kJ mol^–1^, which is based on typical energy differences between polymorphs,^11^ provides good accuracy for the systems studied. Candidates above the uncertainty threshold are added to the training set until either there are no more candidates or the percentage of candidates above the uncertainty threshold is below a specified target. Additionally, a maximum training set size can be set. The scheme is summarized in Figure 2 (top panel).

### On-the-Fly Training

The on-the-fly training scheme we propose here (bottom panel of Figure 2) is based on threshold MC simulations for sampling the configurational space. This method has been applied to molecular crystals using empirical force fields and DFTB to characterize the global structure of crystal energy landscapes^60^ and reduce overprediction of polymorphism.^49^ These simulations involve regular MC sampling of the configurational space, but with the distinguishing feature of an energy lid, which is defined relative to the energy of the initial configuration from which the simulation was initiated. During the simulation, MC moves are accepted if and only if the energy of the resulting structure is below the current energy lid. Consequently, the energy lid effectively constrains the simulation to explore only regions accessible below the lid, thereby providing a high level of control over the trajectory.

With on-the-fly training, each MC step is first evaluated by a cNNP. If the uncertainty of the predicted energy is above the specified threshold, then the step is further evaluated by the reference method, in this case PBE-D3, and the configuration is added to the training set. The cNNP is constantly retrained as structures are added to the training set to ensure reliable uncertainties and to avoid adding redundant structures. For the MC simulations described here, we use a rigid molecule moveset consisting of molecular rotations and translations as well as unit cell lengths, angles, and volume changes. Conformational changes can be added to explore intramolecular perturbations to the crystal structure. Further details of the simulations are provided in the Supporting Information.

## Results

### Optimizing Active Learning for CSP Landscapes

A primary consideration in the development of machine-learning models is determining an appropriate set of hyperparameters. For an MLIP, there are hyperparameters for the model, such as the network architecture of NNPs, as well as hyperparameters for the descriptor, for example, the radial cutoff. Active learning has its own hyperparameters, including the batch size and uncertainty threshold, and also a query strategy. In order to apply active learning efficiently with CSP datasets, we first investigated optimizing the hyperparameters and selection strategy. The aim was to identify the approach that yields the smallest training set that accurately captures the whole landscape, as measured by small test errors, and does so consistently with minimal variation.

The dataset we chose for these studies was a predicted landscape for oxalic acid containing 1965 crystal structures. We chose this landscape because oxalic acid is a known challenging system for empirical force fields^61^ and thus learning either the total energy or Δ-learning the correction from force field to DFT will be meaningful tests. Furthermore, the small size of the oxalic acid structures meant calculating the entire dataset at the target level, PBE-D3, was possible, and thus the results were able to be verified through 5-fold cross-validation.

#### Hyperparameters

The hyperparameters we chose to investigate for optimization were: the target quantity, the committee size, the batch size, and the target percentage of structures above the uncertainty threshold. The results of varying these parameters individually with 5-fold cross-validation are shown in Table 1. In all cases, the uncertainty threshold was set at 1.0 kJ mol^–1^, and structures were added to the training set by highest uncertainty.

**Table 1:** Average MAE, RMSE, and Dataset Size with Standard Deviations from 5-Fold Cross-Validation Given in Parentheses of Combinations of Active Learning Hyperparameters Evaluated by Training cNNPs with an Oxalic Acid CSP Landscape^a^

entry	training quantity	committee size	% uncertain target	batch size	energy MAE (kJ mol^–^^1^)	energy RMSE (kJ mol^–^^1^)	final training set size
1	*E*	6	10.0	30	1.11 (0.05)	1.74 (0.18)	852 (24)
2	*E*, *F*	6	10.0	30	1.08 (0.10)	1.50 (0.13)	546 (40)
3	Δ*E*	6	10.0	30	1.09 (0.11)	1.47 (0.18)	205 (53)
4	Δ*E*, Δ*F*	6	10.0	30	0.92 (0.06)	1.20 (0.07)	216 (22)
5	Δ*E*	6	2.5	30	0.89 (0.11)	1.21 (0.20)	354 (72)
6	Δ*E*	6	5.0	30	0.97 (0.06)	1.29 (0.10)	252 (45)
7	Δ*E*	2	5.0	30	1.19 (0.18)	1.61 (0.26)	168 (65)
8	Δ*E*	18	5.0	30	0.90 (0.06)	1.20 (0.12)	288 (41)
9	Δ*E*	6	5.0	15	1.01 (0.05)	1.37 (0.17)	216 (15)
10	Δ*E*	6	5.0	60	0.90 (0.08)	1.38 (0.43)	320 (45)

a The cNNPs were trained either on total energy (*E*)/forces (*F*) or the difference between the force field (FIT + DMA) values and the reference values (Δ*E*/Δ*F*), i.e., Δ-learning. All entries used an uncertainty cutoff of 1.0 kJ mol^–1^, with candidates selected by highest uncertainty.

The most influential hyperparameter on the final training set is the training quantity (compare entries 1–4, Table 1). Δ-Learning dramatically reduces the size of the training set by up to 76% while achieving similar, if not better, accuracy than learning the total energy (or energy and forces). Importantly, the improvement was similar if restricted to training only on the energy differences without forces, which is expected to be a common application since atomic forces are often not stored with CSP landscapes. However, if atomic forces are available, including them in the training is likely worthwhile and would yield an improved description of the energy surface around the lattice energy minima, which may be important for further calculations beyond the lattice energy correction, for example, calculations of vibrational modes. Compared to the training quantity, the other hyperparameters are less significant, yet tuning these parameters does offer notable improvements, particularly in the efficiency. For example, we found that a large NNP committee of 18 members does not offer significant improvement over a smaller committee of 6 members, despite incurring significantly greater costs. The improved average errors with the 18-member committee are within that expected due solely to a larger committee (Figure S2), suggesting the dataset chosen by active learning is not of higher quality. Moreover, while smaller committees could provide adequate results, they were found to generally underestimate the standard deviation (Table S3) and thus the uncertainty. Similar trends of diminishing returns are observed in the other hyperparameters that were studied. Overall, we identify the parameters of entry 6 as the best balance between accuracy and cost, and we use these settings in the following sections.

#### Query Strategies

We also investigated different strategies for adding candidates to the training set, beginning with comparing active learning random sampling and the highest uncertainty sampling. The former involves evaluating each candidate once, in random order, adding those above the uncertainty threshold to the training set, and retraining when reaching the batch size. By contrast, highest uncertainty sampling, which is the most common strategy for MLIPs, evaluates all remaining candidates at each iteration and adds the candidates with the highest uncertainty to the training set. Additionally, we implemented a strategy combining highest uncertainty sampling and FPS. This strategy, which sampled candidates above the uncertainty threshold by FPS, starting from the candidate with the highest uncertainty, was intended to reduce redundancy in the training set that may arise when sampling by highest uncertainty with a batch size greater than one.

**Figure 3:**
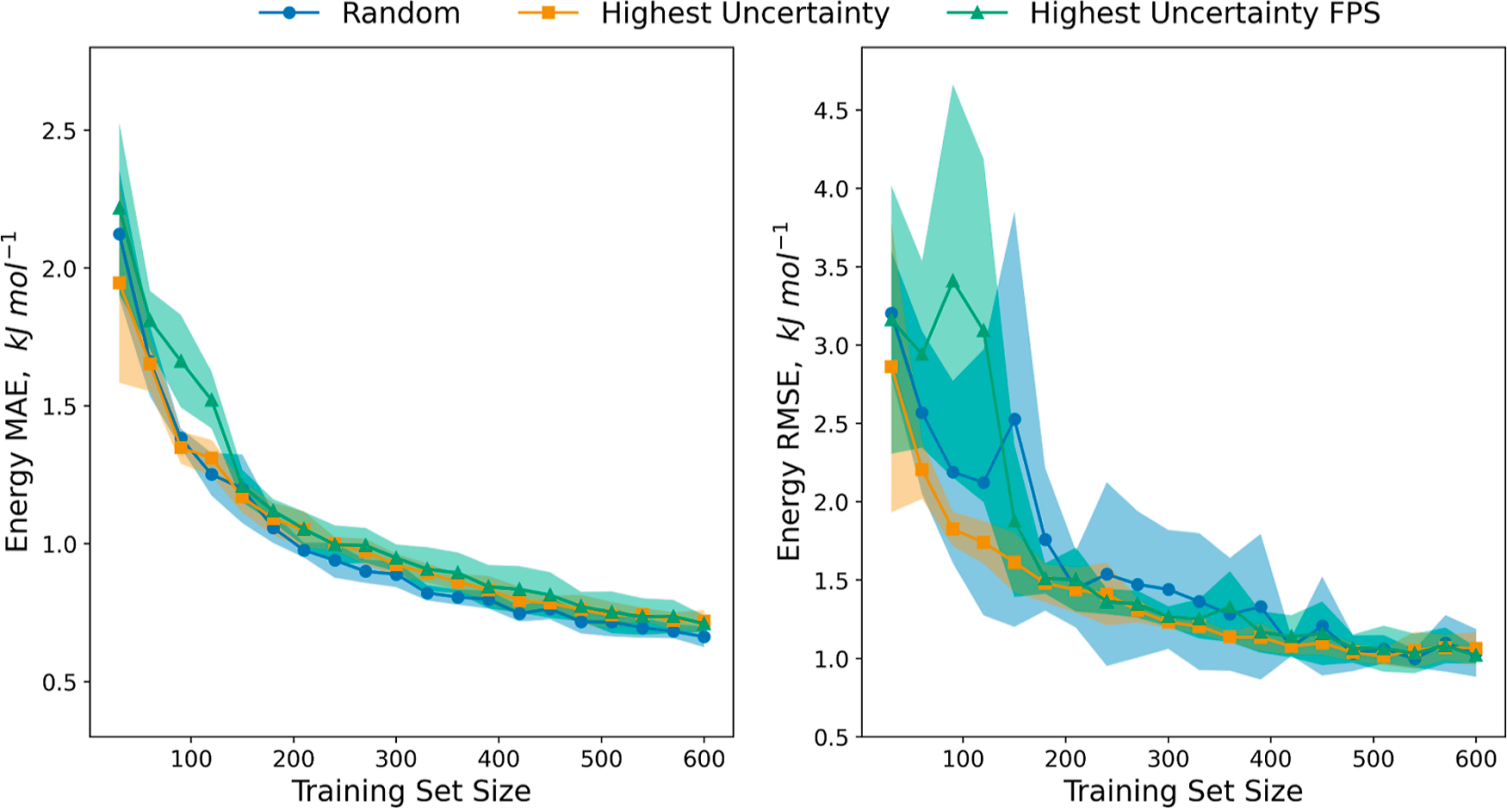
Learning curves in energy MAE (left) and energy RMSE (right) from 5-fold cross-validation for the three strategies. Average values across the 5-folds are indicated by solid lines, while the shaded area represents one standard deviation. Active learning hyperparameters are the same as entry 6 of Table 1, except the uncertainty cutoff was decreased to 0.5 kJ mol^–1^ to extend the active learning to a training set size of 600 across folds.

Comparing these strategies by 5-fold cross-validation, we found their performance to be similar (Table 2). On average, highest uncertainty sampling converged fastest, but the smaller dataset also had on average higher errors than random sampling. The training curves (Figure 3) make the differences between strategies clearer. Here, we found that highest uncertainty sampling had faster convergence with significantly smaller variance as measured by both MAE and RMSE. By contrast, the RMSR from random sampling converged slower and with higher variance even at large dataset sizes. Interestingly, the highest uncertainty FPS strategy did not show improvement over the regular highest uncertainty sampling. This may indicate that the weighting of FPS and highest uncertainty sampling needs adjusting. Nevertheless, the results suggest that there is no significant benefit of the strategy over regular highest uncertainty sampling, which from the oxalic acid results is the best of the three strategies for training cNNPs from CSP datasets.

**Table 2:** Results of 5-fold Cross-Validation for the Active Learning Strategies Evaluated by Training cNNPs with an Oxalic Acid CSP Landscape^a^

strategy	energy MAE (kJ mol^–^^1^)	energy RMSE (kJ mol^–^^1^)	final dataset size
random	0.86 (0.04)	1.20 (0.13)	320 (36)
highest uncertainty	0.97 (0.06)	1.29 (0.10)	252 (45)
highest uncertainty FPS	0.96 (0.10)	1.33 (0.16)	288 (67)

a All cases used the active learning hyperparameters in entry 6 of Table 1 with the uncertainty threshold of 1 kJ mol^–1^.

### Correcting Low-Level CSP Landscapes to Ab Initio Level

Due to the exacting accuracy required, a primary task in organic CSP is correcting lower-level landscapes to higher levels of theory. This may also include reoptimization of the geometries of predicted structures. However, single-point corrections are also common, where the geometries are not updated when energies are re-evaluated at the higher level. The latter correction is especially relevant for MLIPs trained by Δ-learning from the CSP landscapes. To investigate this application, we applied our active learning workflow detailed in the previous section to the CSP landscapes of two challenging systems, TTBI and resorcinol, and then used the resulting training set to train cNNPs to generate corrected landscapes. The final cNNPs consisted of 18 members since these provide slightly better prediction averages over the 6 member committees used in the active learning (Figure S2) and have negligible cost post-active learning.

#### Triptycene-tris(benzimidazolone)

The first application we describe is for TTBI, a triptycene derivative with five known polymorphs and a propensity for forming highly porous, hydrogen-bonded organic frameworks.^50,62,63^ The initial landscape used to train the cNNP was reported by Zhu et al.^50^ and was produced using the FIT + DMA potential, which does an adequate job at identifying the experimental structures and separating them from the bulk of the predicted structures. However, the relative energy differences between the polymorphs are questionable, with the gap between the global energy minimum structure (corresponding to the densely packed ϵ polymorph) and the least dense porous γ polymorph at nearly 100 kJ mol^–1^. Although solvent incorporated in the voids was shown to stabilize the porous polymorphs during growth,^63^ the FIT + DMA polymorph energy differences seem unreasonably large. The relative energies are also sensitive to the calculation method: DFTB-D3 optimization reduces the energy gap between the polymorphs,^50^ as do predictions using a different (W99 + DMA) force field.^63^ Understanding the achievable energetic range for metastable crystal structures with attractive properties is important for developing the use of CSP for material discovery. However, calculating higher-level energies for such CSP landscapes has thus far been too computationally expensive due to both the large size of the structures and the scale of the landscape: the TTBI CSP landscape used here contains 14,997 distinct structures. Furthermore, the landscape exhibits a diverse range of structures covering a very wide density range, from primarily dispersion-bound structures to hydrogen-bonded structures: this diversity in intermolecular interactions is a further challenge to training a MLIP to predict accurate energies.

Considering the results of our hyperparameter and strategy tests, we performed active learning with a committee of 6 NNPs, training on Δ*E*, and adding structures by highest uncertainty. Due to the larger size of the structures (46 atoms/molecule), the uncertainty threshold was set at 2 kJ mol^–1^ per molecule, and the batch size was set to five structures. Furthermore, to focus the potential toward the lower-energy structures, we applied a cutoff at 110 kJ mol^–1^ above the global energy minimum, which gave 2220 candidate structures for training and included all matches to the experimental polymorphs.

**Figure 4:**
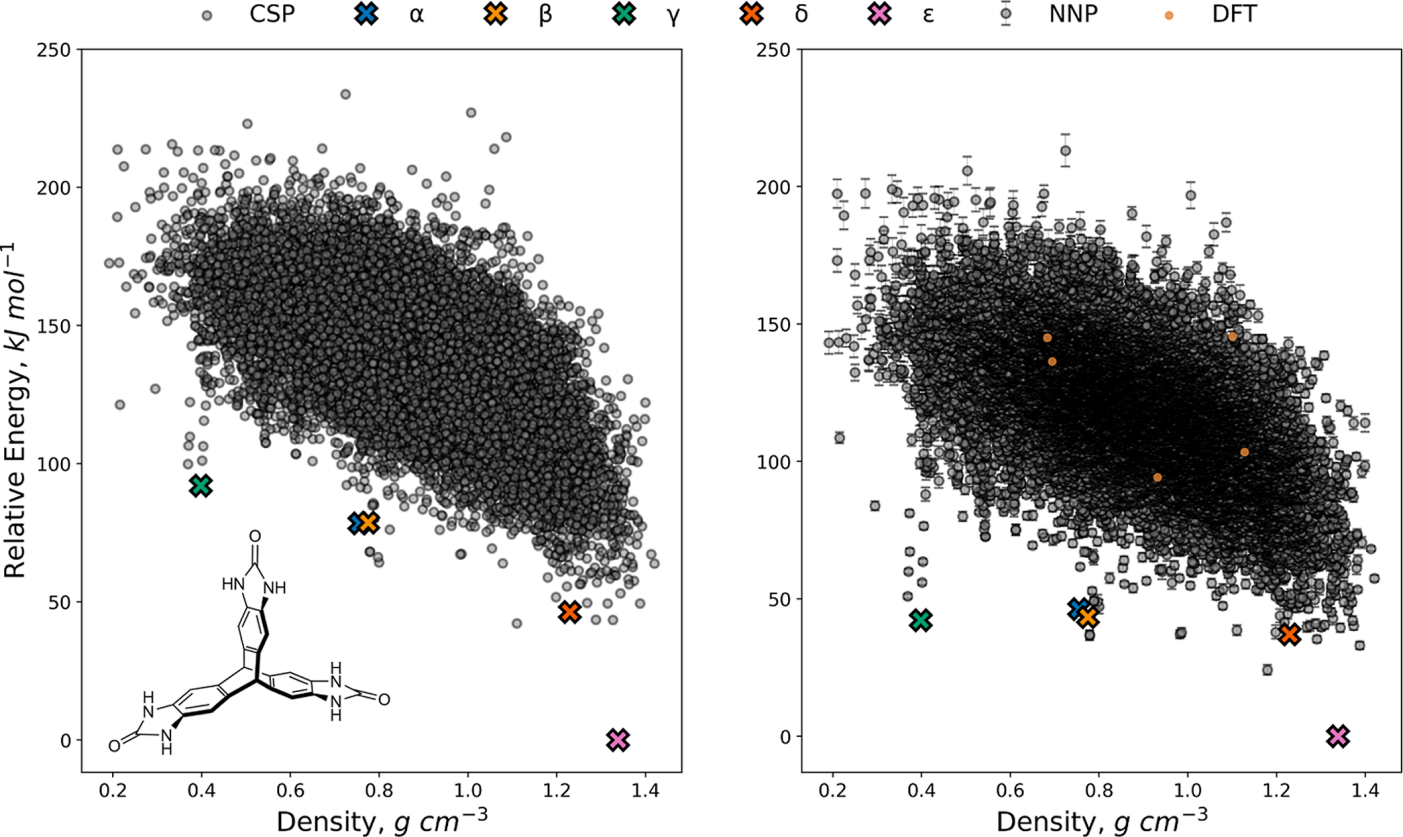
FIT + DMA landscape (left) and cNNP reranked landscape (right) for TTBI. Error bars on cNNP energies correspond to one standard deviation in the committee predictions. Structures with energies beyond 250 kJ mol^–1^ above the global minimum have been omitted for clarity. Structures marked with an X correspond to experimentally observed polymorphs. Yellow markers indicate structures with high uncertainty (>6 kJ mol^–1^) that were evaluated directly with the target method.

With these settings, the active learning converged in 185 structures, corresponding to less than 10% of the candidates and only 1.2% of the total landscape. The corrected landscape calculated with the final potential is presented in Figure 4, with the uncertainties represented by error bars on each structure. Despite the energy cutoff and small training set, the potential achieves good accuracy across the entire landscape: only 9 out of the 14,997 structures had uncertainties above 6 kJ mol^–1^. The energies of these structures were computed directly with PBE-D3. Pleasingly, the correction yields a considerable reduction in the energy range of the experimentally observed polymorphs, with the gap between the global minimum and the low density (α, β and γ) polymorphs reducing to less than 50 kJ mol^–1^, which is in line with the solvation stabilization estimated for these structures.^64^ Comparing the corrected energies to calculated PBE-D3 energies for 92 of the lowest-energy structures on the initial landscape (16 of which were selected by active learning), we find a MAE of 3.1 kJ mol^–1^ and a RMSE of 4.1 kJ mol^–1^ (Figure S3). In terms of energy rankings, following the correction, all five known polymorphs are ranked in the 30 lowest-energy structures, with the biggest change in rank being observed for the very low density γ polymorph, which dropped from 647th to 21st on the landscape. This remarkably good ranking of the polymorphs with such a minimal training set is highly encouraging for the application of the active learning workflow to other diverse, large-scale landscapes and highlights the advantage of the correction even for landscapes where the low-level method is initially thought to perform reasonably well.

#### Resorcinol

We next investigated applying the active learning workflow to resorcinol, a small organic molecule that has been well-studied as an example of conformational polymorphism. The initial CSP landscape, which was calculated from a pool of conformations and relaxed at the DFTB-D3 level, contains matches to the observed α and β polymorphs. The molecules in these polymorphs differ conformationally by rotating one hydroxyl group 180°, transforming between the *syn*–*syn* and *syn*–*anti* conformations. This conformational flexibility means that, whereas the models trained for oxalic acid and TTBI were effectively intermolecular potentials, correcting the resorcinol landscape requires training a model that describes both intramolecular and intermolecular energy corrections. To add to this, the DFTB-D3 description of the relative energies of the resorcinol structures contains clear deficiencies and correlates poorly with the target PBE-D3 relative energies (Figure S2), which increases the difficulty in learning the correction. It is also notable that the DFTB-D3 landscape incorrectly ranks the β polymorph lower in energy than the α polymorph, opposite to the expected order.

The active learning was performed with the same settings as for TTBI, except in this case, the uncertainty threshold was set to 1.0 kJ mol^–1^, and the batch size was set to 15 structures. A cutoff at 65 kJ mol^–1^ above the global minimum was applied, resulting in a candidate pool of 2487 structures, which contained matches to both the α and β polymorphs.

**Figure 5:**
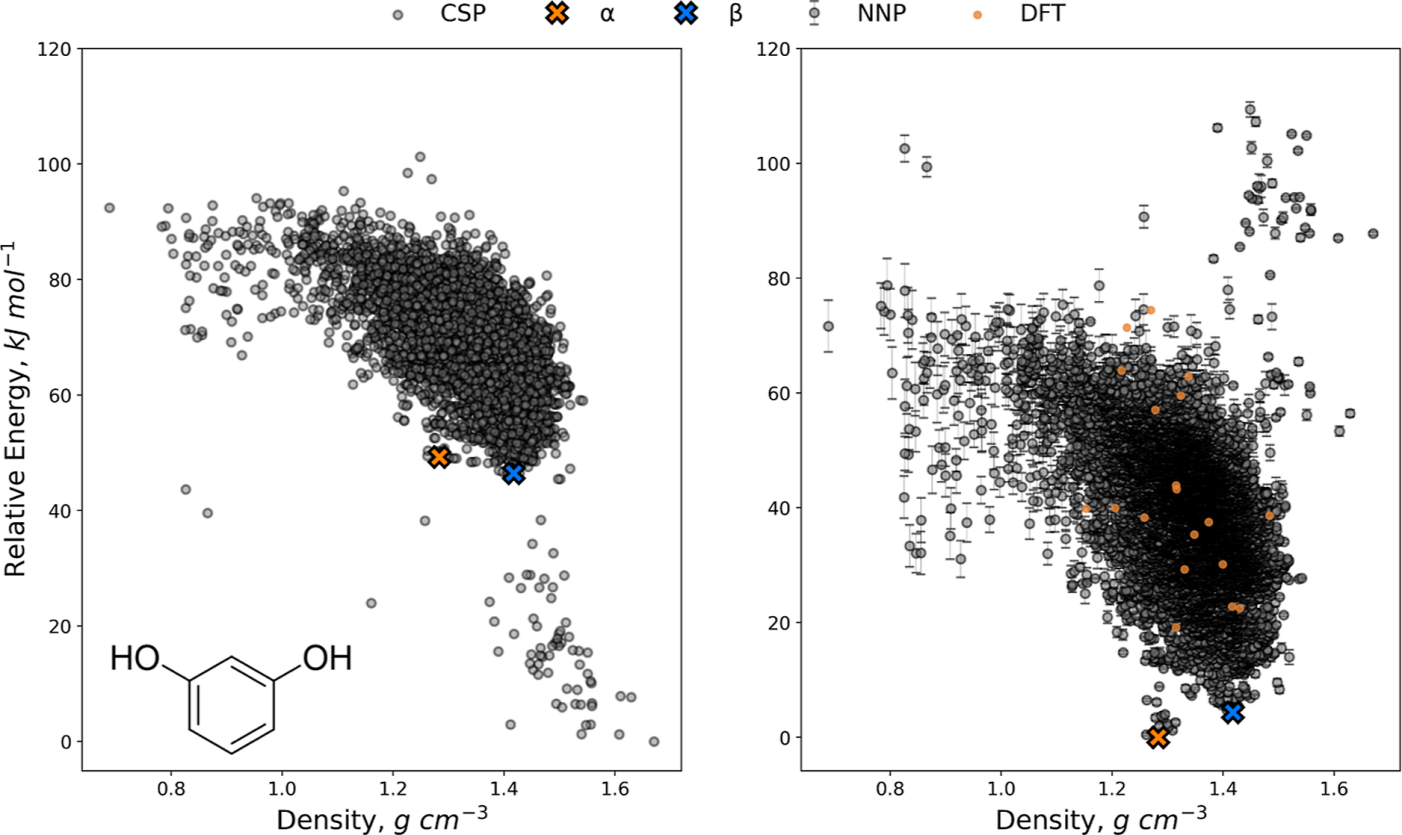
DFTB-D3 landscape (left) and cNNP reranked landscape (right) for resorcinol. Error bars on cNNP energies correspond to one standard deviation in the committee predictions. Structures marked with an X correspond to experimentally observed polymorphs. Yellow circles indicate structures with high uncertainty (>6 kJ mol^–1^), which were evaluated directly with the target DFT method.

With these settings, active learning was completed after adding 780 structures, i.e., 31% of the candidates and 9% of the total landscape. This is significantly higher than that seen for oxalic acid or TTBI, illustrating the impact of a poor correlation between the baseline (DFTB-D3) and target (PBE-D3) methods, which increases the complexity of the function that the model is attempting to fit. The corrected landscape evaluated with the final potential is presented in Figure 5. Out of the 8808 structures, 19 had uncertainties above 6 kJ mol^–1^ and were evaluated directly by the target method. Examining these structures, most were only slightly above the threshold, and the predicted energies were close to the computed energies, suggesting that the cutoff at 6 kJ mol^–1^ may have been tighter than needed.

Comparing the corrected with the initial DFTB-D3 landscapes, we can see many of the deficiencies from the baseline (DFTB-D3) landscape have been eliminated. The structures corresponding to the experimentally observed polymorphs are now among the lowest-energy structures on the landscape and in the correct expected order of stability, with the α polymorph being the global minimum and the β polymorph ranked slightly higher. The CSP structures that were predicted with low energies and high densities by DFTB-D3 have been corrected to higher energies and are now the highest-energy structures on the landscape, emphasizing the very poor description of these structures on the initial landscape. Despite the large correction required, for the vast majority of the landscape, the potential estimates the corrections with low uncertainty, with the mean uncertainty being 1.51 kJ mol^–1^. Moreover, comparing the corrected energies to the calculated PBE-D3 energies for the 300 lowest-energy structures on the initial landscape (106 of which were selected by active learning), we find a MAE of 0.4 kJ mol^–1^ and a RMSE of 0.6 kJ mol^–1^ (Figure S2). The only notable exceptions are the low-density CSP structures, which have larger uncertainties. This is due to these structures being some of the highest-energy structures on the initial landscape, and thus relatively few ended up in the set of candidates for active learning. Nevertheless, despite not training on many of these low-density structures, considering the energy range of the landscape, the uncertainties are not excessive. Overall, the potential has performed exceedingly well considering the difficulty of the landscape and has notably succeeded in learning the combined intramolecular and intermolecular corrections to a high standard.

### On-the-Fly Training

The potentials so far presented have been trained exclusively on the energy minima of precomputed CSP landscapes and consequently have a limited description of the energy surface beyond these points. Here, we look at how we can improve the description of the PES through on-the-fly training within MC simulations. We demonstrate this using the 300 lowest-energy structures from the CSP landscape predicted for oxalic acid.

Before we can begin the simulations, we first need to determine which structures to sample with the MC trajectories. Ideally, we want to select structures that are diverse and well-separated on the energy surface, such that the simulations cover as much of the energy surface as possible with the fewest number of trajectories. Redundant structures that occupy similar regions of the energy surface will add little to improving the MLIP while increasing the computational cost. The area of the energy surface that will be covered by each trajectory is difficult to determine a priori; however, using FPS, we can ensure that our selected structures represent a diverse set. Thus, we selected 10 structures from the set of 300 oxalic acid structures by FPS in the descriptor space starting from the lowest-energy structure, which also matches the β polymorph. On-the-fly training from these structures with an uncertainty threshold of 2.0 kJ mol^–1^ yielded 1636 structures from the MC trajectories that were added to the training set.

To illustrate the improvement of the potential, we first ran simple downhill MC simulations on the 300 oxalic acid structures (Figure S4). These simulations, which only accept MC moves that decrease the energy, are relatively localized and constrained effectively to the initial energy basin. Nevertheless, using the initial cNNP trained on CSP minima, we find that only 9 CSP structures remain stable after 1500 MC steps. The other 291 trajectories were terminated early due to high uncertainties in energy predictions in excess of 50 kJ mol^–1^. By contrast, performing the same simulations with the on-the-fly trained cNNP 299 of the 300 trajectories remain stable.

**Figure 6:**
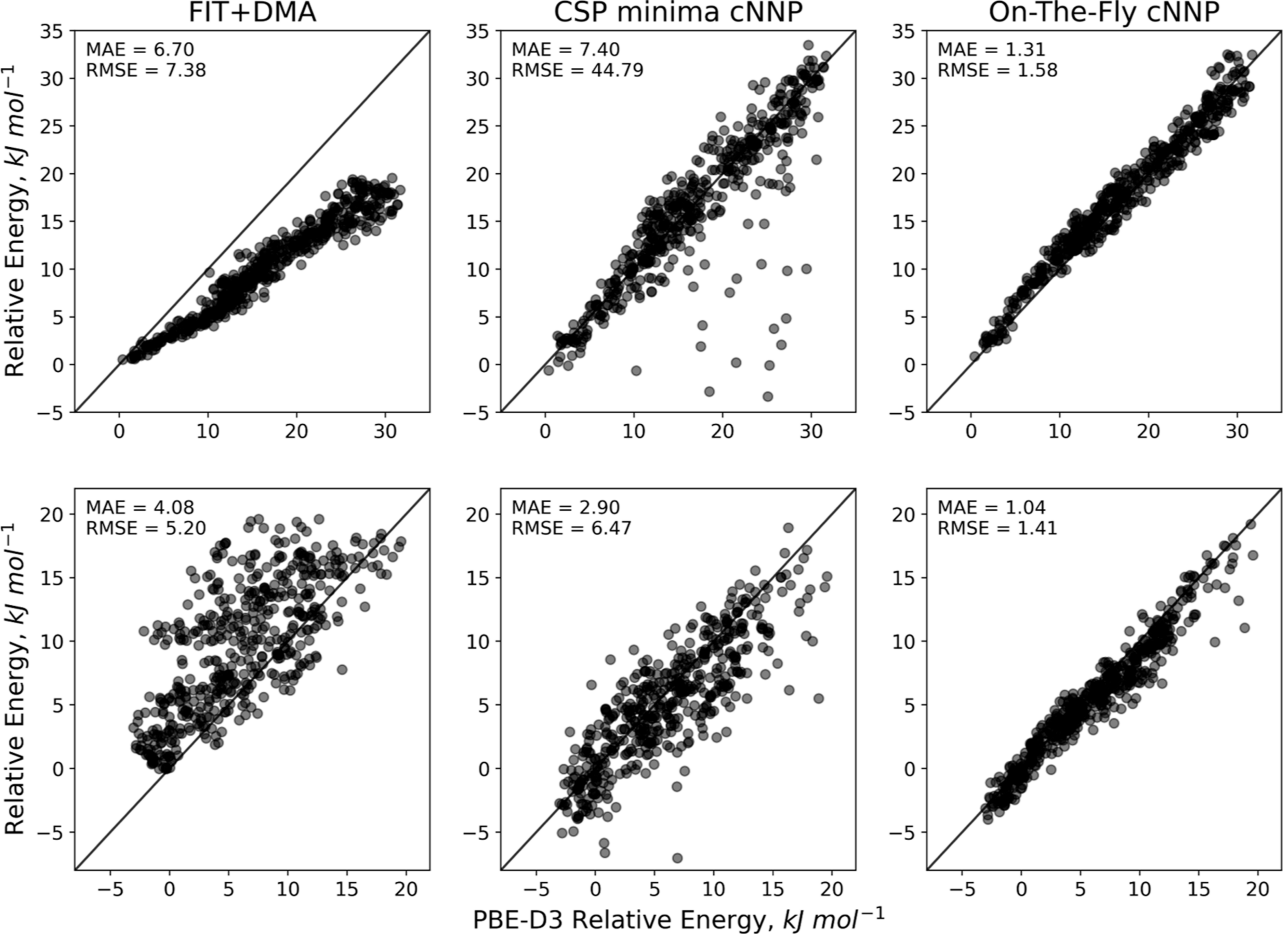
Correlation of FIT + DMA, CSP-trained cNNP, and the CSP-trained cNNP with additional MC on-the-fly training with the PBE-D3 reference for a set of unminimized accepted structures sampled from FIT + DMA MC trajectories of the α (top) and β (below) polymorphs of oxalic acid.

To further qualify the differences in the potentials, we generated a test set of 1000 unminimized structures randomly sampled from FIT + DMA MC simulations of the α and β polymorphs. The MC simulations sampled an energy up to 20 kJ mol^–1^ above the initial energy and were sampled evenly, such that 500 structures were from the α polymorph trajectory and 500 were from the β polymorph trajectory. The correlations of energies for these structures calculated by PBE-D3 against those calculated by FIT + DMA, the cNNP trained on CSP minima, and the cNNP with on-the-fly training are shown in Figure 6. Considering first FIT + DMA, there is a notable and pronounced systematic underestimation of the energies for the α polymorph structures and yet simultaneously a systematic overestimation for the β polymorph structures. These inconsistent errors reflect the difficulty in accurately capturing the oxalic acid energy surface with the simple functional form and thus emphasize the limitations of the FIT potential for this system.

By contrast, the cNNP trained on CSP minima does not exhibit similar systematic errors and, for most structures, achieves low errors. Indeed, for the structures sampled from the β polymorph trajectory, the model achieves a significantly lower MAE than FIT + DMA. However, the limitation of this model and the cause of the unstable downhill MC trajectories are a small number of structures that the model returns excessively large errors for, often more than 100 kJ mol^–1^. The effect of these outliers can be seen in the RMSEs, which are multiple times larger than the MAEs. Pleasingly, the uncertainties of the outlier predictions are similarly large. For example, removing structures with uncertainties above 10 kJ mol^–1^ for the α trajectory structures, which correspond to 72 structures (from 500 total), we find the underlying MAE and RMSE to be 1.56 and 2.03 kJ mol^–1^, respectively. Therefore, while the extrapolation of the model is better than expected for the majority of structures, highlighting the broad relevance of potentials trained on CSP minima, the prevalence of outliers suggests an incomplete description of the energy surface.

Improving this description is the aim of the on-the-fly training, and comparing the correlations before and after the on-the-fly training, there is a clear improvement. Not only are all outliers eliminated but also the errors across the test set are significantly reduced, resulting in notably lower MAEs. Considering the α polymorph was not among the initial structures in the on-the-fly training, the improved accuracy for these structures is particularly impressive. For comparison, if we do include an on-the-fly simulation initiated from the α polymorph, the resulting model achieves a MAE of just 0.5 kJ mol^–1^ on the same structures (Figure S5). The possibility to further improve the accuracy with further sampling is also evident in the subtle trend of larger errors at higher energies, which suggests that the sampling at these energies could be insufficient. However, this is not unexpected considering that the on-the-fly training trajectories were shorter than the trajectories the test structures were sampled from and, moreover, may have sampled different regions due to the different energy models.

Beyond accuracy, we were also interested in improving the efficiency of the on-the-fly sampling, which seemed achievable considering the generally good accuracy of the cNNP trained on CSP minima, implying that only a small number of structures with high uncertainties need to be added to the training set to yield a robust potential. To investigate this, we repeated the first on-the-fly training, starting from the same 10 structures but with a higher uncertainty threshold of 10 kJ mol^–1^. This change resulted in only 91 structures being added to the training set, a reduction of 95% compared to on-the-fly training with a 2 kJ mol^–1^ threshold. Despite the smaller training set, the model performs well. The correlation plots of this model (Figure S6) show that it still achieves a significant improvement compared to the model trained only on CSP lattice energy minima and, importantly, eliminates the outliers, which suggests a robust description of the energy surface. Of course, the average errors are not as small as with the 2 kJ mol^–1^ uncertainty threshold. However, considering the reduced computational cost, it could be a worthwhile compromise, allowing longer, higher energy trajectories and/or more trajectories during the on-the-fly training.

## Discussion

The diversity of structures typically found on organic CSP landscapes provides a great resource for training MLIPs that are relevant across a wide range of the lattice energy surface. The results presented here demonstrate how active learning combined with Δ-learning provides an efficient workflow to generate MLIPs from these datasets. The resulting potentials can be applied directly to correct the energies of CSP structures to higher levels of theory or extended by on-the-fly training within MC simulations to accurately describe the energy surface beyond the local energy minima.

The presented workflow should be generally useful for organic CSP and can help address the often prohibitive costs associated with the DFT ranking of predicted structures.^10^ For instance, training to the TTBI landscape was completed at a cost of 1498 CPU hours, which is conservatively estimated to be a 155-fold reduction compared to evaluating the landscape directly at the target (PBE-D3) level of theory. In real time, this corresponds to the difference between 20 h and 130 days using 80 Intel(R) Xeon(R) Gold 6248 CPU cores@2.50 GHz. Similar efficiency was observed for resorcinol, with the cost of the corrected landscape estimated at 1293 CPU hours. The on-the-fly sampling is also notably efficient. The initial sampling, which added 1636 structures to the training set, corresponded to only 3.6% of the structures evaluated during the simulations. However, as shown in the results, if willing to accept lower accuracy, a similarly robust potential can be achieved with considerably less sampling and thus a lower cost.

These results focused on achieving a first approximation of the DFT landscape, which is a common part of organic CSP workflows. For some systems, higher-level rankings, including free energy corrections, are important.^65^ In such cases, low-energy structures from the MLIP-corrected landscape can be selected for these calculations, as is typically done. However, with further training, such as on-the-fly training including atomic forces, we envisage the MLIPs themselves could be used for these calculations. MLIPs that accurately predict vibrational spectra have been demonstrated in other studies, and thus we are reasonably confident the MLIPs we have presented could be extended to high-level rankings of organic crystals, including free energies.

The workflow developed here is applied to a precomputed CSP landscape. Therefore, the methodology can be applied to existing legacy or published landscapes as well as new CSP studies. However, the requirement for a precomputed CSP structure set means that a “good enough” baseline model is required. Where the baseline is an empirically parametrized force field, molecules with less common functional groups or elements might be less well modeled by common empirical force fields; therefore, either developing tailored force field parameters or using higher-level and likely more expensive methods, such as DFTB, might still be required to generate the initial landscape. A further consideration is that the potentials generated with the methods presented here are reliable at local minima on the lattice energy surface and, when on-the-fly training to MC trajectories is included, are accurate in the local region of the lattice energy surface. Thus, as shown in the on-the-fly training results, the potentials can achieve lattice energy minimizations from good starting structures. However, properties and behaviors that require a broader description of the lattice energy surface, such as transitions between polymorphs, might require the potential to extrapolate beyond its training, so risks loss of accuracy.

An alternative approach, which addresses both issues, would be to train the MLIP on-the-fly at the structure-generation stage of CSP so that the training sees high energy configurations and can correct for deficiencies in the force field while the landscape is being generated. This type of approach has been demonstrated for inorganic CSP,^25,29^ where the CSP search is frequently performed at the ab initio level, and so there is a stronger impetus to improve efficiency in this stage. Due to the large range of interactions explored in the CSP of organic molecules, we expect that a similar approach would result in much larger training datasets relative to what is needed when aiming to model the lattice energy minima and their local regions.

Beyond the scope of the MLIPs, the variability in the active learning results is also notable. As shown in the results for oxalic acid, even when using the optimal parameters and strategy identified, we found significant variation in the training sets selected. This is especially clear when the active learning was repeated while keeping all parameters except the starting structures constant (Table S2). While ML models have inherent variability due to stochastic elements involved in training, considering that the most expensive part of developing MLIPs is typically in generating the reference data, minimizing variation in the selected training set should be a priority and is worth further study.

Another area for future development is to automatically partition the lattice energy into intramolecular and intermolecular contributions to improve the model’s applicability to flexible molecules. Our results for resorcinol illustrate that a single model can accurately capture the intramolecular and intermolecular components of a landscape with limited conformations. However, other studies have found that the difference in scale between inter- and intramolecular interactions means that capturing both with a single model is often limiting and that training separate intermolecular and intramolecular models yields improved performance.^46,66^ Partitioning the energy will also make applying the workflow over multiple landscapes more practical, which could allow for training transferable rather than system-specific models. The development of universal models for organic molecules^38,67−70^ and inorganic materials,^71−73^ has produced impressive results with good transferability; similar models for organic crystals could have an important impact in the field of CSP.

## Conclusions

Computational efficiency is an important aspect of crystal structure prediction and its practical applications. As seen in the recent blind tests, the increasing use of high-level quantum chemistry calculations for correcting initial CSP landscapes has led to dramatic increases in computational costs. Notably, these increasing costs are causing a disparity between researchers and groups that have access to large-scale computational resources and those that do not and so limits the impact of these methods in polymorph screening, crystal engineering, and material discovery. In this context, accurate MLIPs have arrived with fortuitous timing and with the potential to reduce the cost of organic CSP without compromising the necessary high-level accuracy.

The workflow we have presented here is a further step toward integrating MLIPs into organic CSP. By combining active learning and Δ-learning, leveraging the lower-level energies describing the landscapes, which are available at no added computational cost beyond the crystal structure search, we have demonstrated a highly efficient and automatable method for generating MLIPs from CSP landscapes. As shown for oxalic acid and resorcinol, active learning from a force field or DFTB baseline can achieve errors at or below 1 kJ mol^–1^, using approximately 10% of the landscape for training. We converged active learning at errors of 3–4 kJ mol^–1^ as being acceptable over a much broader energy range of predicted crystal structures, using only 1.2% of structures for training.

Furthermore, we illustrated how these potentials can be readily extended to points on the lattice energy surface far from the initial CSP structures through on-the-fly training within MC simulations. The resulting potential yielded stable crystal structure optimizations. Future studies will investigate training separate models for the intramolecular and intermolecular components toward an improved description of conformationally flexible systems, applying transfer learning and multifidelity approaches to reach higher levels of theory efficiently, and a more advanced training scheme to reduce variability in the active learning. Our results here further exemplify the potential of MLIPs to accelerate organic molecular CSP, and with the improvement in MLIP models and descriptors ongoing, there is still much more to be realized.

## Data Availability

The CSP datasets, training sets, and final cNNP parameters for oxalic acid, TTBI, and resorcinol are available at https://doi.org/10.5258/SOTON/D2840. Python code implementing the active learning strategies is available at https://github.com/pwvbutler/CSP-AL.

## Supporting Information Available

Details of crystal structure prediction methods, NNPs, DFT calculations and on-the-fly MC simulations; further results of active learning and energy correlations for oxalic acid, resorcinol and TTBI; detailed on-the-fly training results(PDF)
