# Supplementary material for: Machine-Learned Potentials by Active Learning from Organic Crystal Structure Prediction Landscapes
Source: J Phys Chem A. 2024 Jan 26;128(5):945–57. doi: 10.1021/acs.jpca.3c07129 (PMC10860135; doi:10.1021/acs.jpca.3c07129)
Supplement: Supplementary file 1 — jp3c07129_si_001.pdf [file jp3c07129_si_001.pdf]

Machine Learned Potentials by Active Learning from Organic Crystal Structure Prediction Landscapes: Supporting Information

Patrick W. V. Butler[a], Roohollah Hafizi[a], Graeme M. Day[a*]

[a]School of Chemistry, University of Southampton, Southampton, SO17 1BJ, UK

[*]Corresponding author: g.m.day@soton.ac.uk

Contents

S1 General Methods **S3**
S1.1 Crystal Structure Prediction . . . S3
S1.2 Neural Network Potentials . . . S3
S1.3 DFT calculations . . . S3
S1.4 On-The-Fly Monte-Carlo Simulations . . . S4

S2 Active Learning **S5**
S2.1 General vs Tailored Descriptor . . . S5
S2.2 Variation . . . S5
S2.3 Committee Size and Standard Deviations . . . S5

S3 Correlations of Models with Target Method **S6**
S3.1 Oxalic Acid . . . S6
S3.2 Resorcinol . . . S7
S3.3 TTBI . . . S8

S4 On-The-Fly Training **S9**
S4.1 Downhill Monte Carlo Simulations . . . S9
S4.2 On-The-Fly Sampling including trajectory from the α polymorph of Oxalic Acid . . . S10
S4.3 On-The-Fly Training with 10.0 kJ mol^{-1} Uncertainty Threshold . . . S11

References **S12**

S1 General Methods

S1.1 Crystal Structure Prediction

The initial CSP landscapes were generated using the GLEE (Global Lattice Energy Explorer) code.[1] For oxalic acid the rigid body scheme described in the Case et al. was used. The same scheme was used in Zhu et al for the initial TTBI landscape. The method is based on rigid-body lattice optimisations using an empirically parametrised intermolecular atom-atom exp-6 potential combined with atomic multipole electrostatics, the parameters sourced from the FIT[2] force field. The entire model is refered to as FIT+DMA. The molecular geometries are optimised at the B3LYP/6-311G(d,p) level using Gaussian09[3] and held fixed through the rest of the search. Distributed, atom-centered multipoles up to hexadecapole are derived from the electron density by a distributed multipole analysis and partial charges fitted to the multipoles.[4,5] Selected space groups are then searched separately using a quasi-random method and valid structures lattice energy minimised using the software packages PMIN[6] and DMACRYS[7] in a 3-stage protocol consisting of: PMIN at ambient pressure with partial charge electrostatics, FIT+DMA at 0.1 GPa with partial charges, and lastly the FIT+DMA once more at ambient pressure with multipole electrostatics. In the case of oxalic acid the 64 most common space groups for organic crystals were sampled with one molecule in the asymmetric unit and the search terminated after at least 5000 valid structures were generated in each space group.

In the case of resorcinol, the CSP landscape was generated by applying our recently developed flexible-molecule CSP protocol.[1] This protocol is largely similar to that described for rigid systems, however, rather than searching the lattice packing space of a single conformation we instead search with a pre-calculated pool of rigid conformations. Structures are then generated by randomly selecting a conformation from the pool. For resorcinol, the pool was generated by fixing one of the hydroxyl group torsions in an anti position while stepping the other through 360 degrees in 40 degree increments. The 25 most common space groups were then searched generating 10,000 valid structures in each. These were lattice energy minimized, initially by the same 3-stage protocol, then after clustering the unique structures were further optimised to allow the conformations to relax in the crystal with D3 dispersion-corrected density functional based tight binding (DFTB+D3) as implemented in DFTB+[8] using the 3ob parameter set.[9]

S1.2 Neural Network Potentials

All neural network potentials reported in this study were produced using the N2P2 code. The architecture of the neural networks consisted of two hidden layers of 25 nodes each, with softplus activation functions on the hidden nodes and a linear activation function on the output node. The Nguyen-Widrow weights scheme was found to improve training without loss of accuracy in estimated uncertainties from committee models. A train-validation split of 90:10 was used throughout. Input files containing the full settings and symmetry functions for each system are included supporting data.

S1.3 DFT calculations

All periodic DFT calculations were performed using the PBE using the PBE exchange correlation functional with the D3(BJ)[10] dispersion correction (PBE-D3) as implemented in VASP[11–14]. In all cases, this was the target level of theory for the NNPs. A plane wave basis set with a 600 eV cutoff was used for oxalic acid and resorcinol while a 500 eV cutoff was used for TTBI. For all calculations a regular k-point grid with density of at least 0.05 Å^{-1} was used. For TTBI and oxalic acid the intramolecular energy was

[1]Manuscript in preparation

removed by calculating the energy of the isolated, rigid molecule using the same method in a cell with a minimum vacuum between periodic images of 20 Å in all directions.

S1.4 On-The-Fly Monte-Carlo Simulations

Threshold Monte Carlo simulations were performed using the implementation within our in-house code cspy with adaptions for on-the-fly training. The oxalic acid simulations conducted used a moveset consisting of molecular translations and rotations as well as unit cell changes including lattice cell lengths, angles and volume changes. At each step one move is randomly selected based with the probability of each move scaled to the proportion of the total degrees of freedom it represents. The magnitude of the selected move type is randomly chosen from the interval specified for that move type. In on-the-fly training the move is first evaluated by the cNNP, if it is below the uncertainty threshold the energy is assumed to be accurate. If it is above the uncertainty threshold a DFT calculation is performed and the result added to the training set. The move is then accepted or rejected based on whether the calculated energy for the move is below or above the current energy lid, respecitively. A process separate from the trajectories continuously checks the training data, retraining the cNNP if the dataset has increased beyond a specified batchsize since last trained.

For the oxalic acid simulations the batchsize was set at 2 structures. The on-the-fly simulations all consisted of 5000 steps, the energy lid starting at 0.5 kJ mol^{-1} above the initial configuration and increasing by 0.5 kJ mol^{-1} every 100 steps. The FIT+DMA trajectories, from which test structures were sampled, were ran for 20,000 steps the energy lid also starting at 0.5 kJ mol^{-1} above the initial configuration and increasing by 0.5 kJ mol^{-1} every 500 steps. The initial on-the-fly training simulations were conducted with an uncertainty threshold of 2.0 kJ mol^{-1}. The test set was sampled from the accepted structures of the FIT+DMA trajectories.

The structures for the initial on-the-fly simulations were selected by farthest point sampling within the symmetry function descriptor space of the 300 lowest energy predicted oxalic acid structures, starting from the global minimum, i.e. the β polymorph.

S2 Active Learning

S2.1 General vs Tailored Descriptor

Table S1: Results from 5-fold cross-validation for active learning from the oxalic acid landscape with a general set of symmetry functions and with CUR selected symmetry functions. The settings from table 1 entry 6 were used in both cases.

Descriptor	Energy MAE (kJ mol^{-1})	Energy RMSE (kJ mol^{-1})	Final Dataset Size
General	0.91 (0.10)	1.28 (0.12)	612 (72)
CUR selected	0.97 (0.06)	1.29 (0.10)	252 (45)

S2.2 Variation

Table S2: Variation of the active learning results over 12 repeats using each of the strategies. The settings from Table 1 entry 6 were used in all cases with the only variation allowed being the initial structures selected. Thereafter structures were selected according to the strategy.

Strategy	Energy MAE (kJ mol^{-1})	Energy RMSE (kJ mol^{-1})	Final Dataset Size
Random	0.83 (0.05)	1.22 (0.12)	325 (34)
Highest Uncertainty	0.96 (0.07)	1.40 (0.14)	251 (48)
Highest Uncertainty FPS	0.85 (0.07)	1.25 (0.19)	365 (83)

S2.3 Committee Size and Standard Deviations

Table S3: The final dataset size and average standard deviation of energy predictions across the entire oxalic acid dataset for cNNPs with various size committees. The trend of increasing dataset set size with increasing committee size seems to be due to smaller committees underestimating the true standard deviation of the predictions. This relationship however is not linear and the underestimation becomes small with moderately sized committees.

Committee Size	Final Dataset Size	Ave. Energy Std. Dev. (kJ mol^{-1})
2	168 (65)	0.36
6	252 (45)	0.61
18	288 (41)	0.67

S3 Correlations of Models with Target Method

S3.1 Oxalic Acid

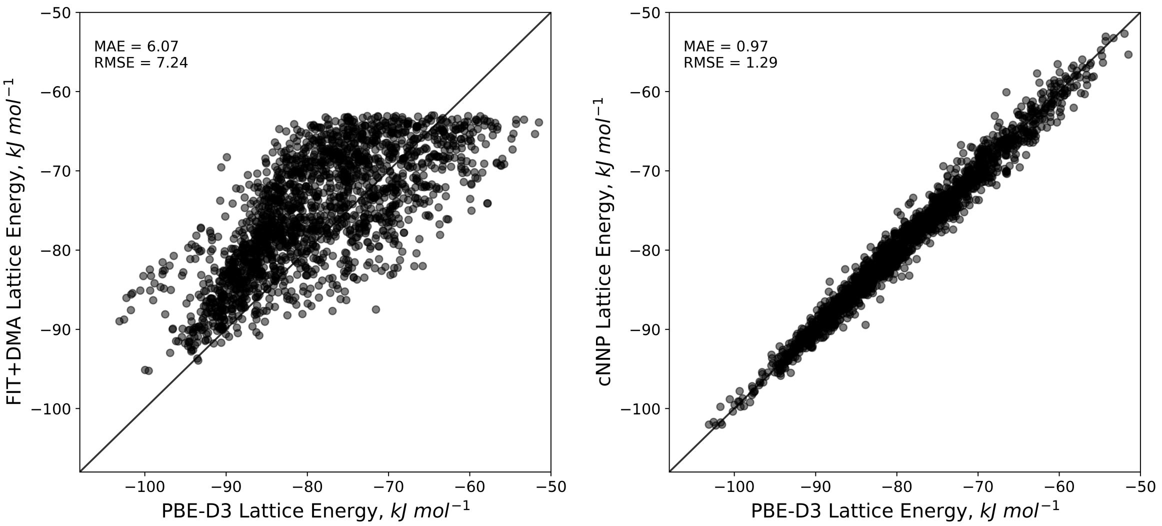

Figure S1: Correlation of FIT+DMA (left) and cNNP test folds (Table 1 entry 6, right) with PBE-D3 for the entire CSP landscape used in the active learning.

S3.2 Resorcinol

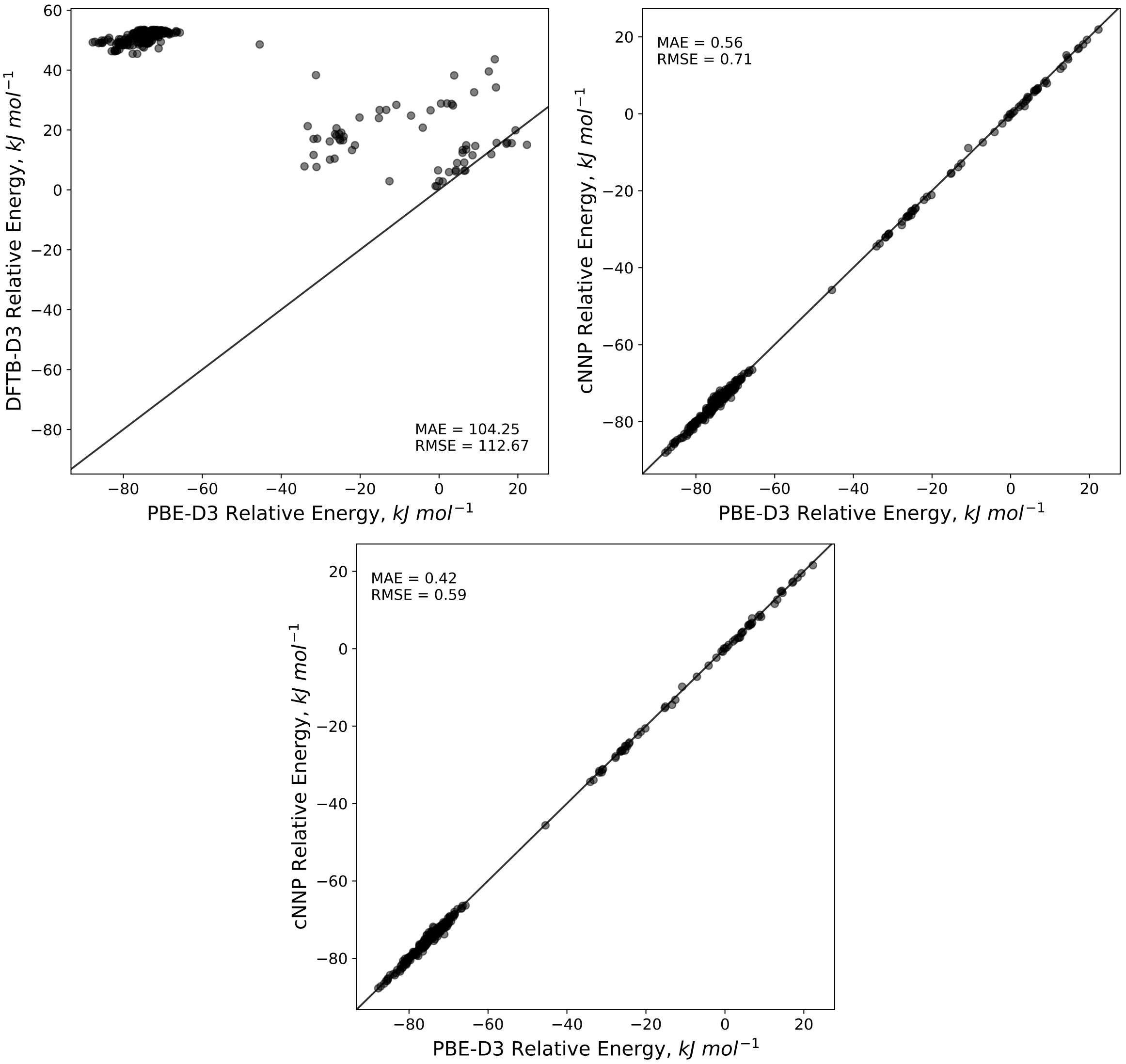

Figure S2: Clockwise from top left: correlation of PBE-D3 energies for a test set of 300 low energy resorcinol structures from the initial landscape with DFTB-D3, the active learning final 6 member cNNP, and the final 18 member cNNP. Energies are relative to the lowest DFTB-D3 energy. Note 106 of the structures were selected by active learning and included in the training set of the cNNPs.

S3.3 TTBI

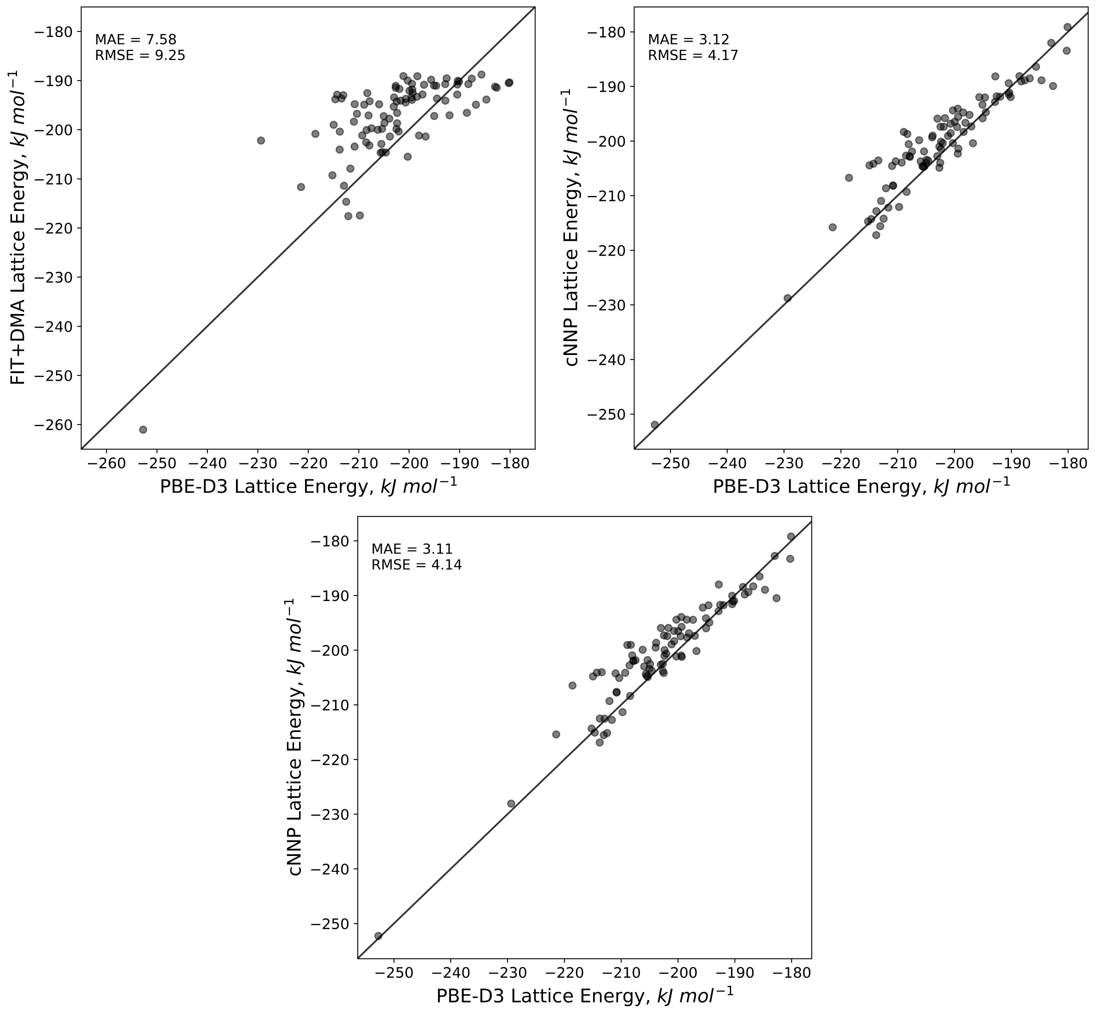

Figure S3: Clockwise from top left: correlation of PBE-D3 energies for a test set of 92 low energy TTBI structures with FIT+DMA, the active learning final 6 member cNNP, and the final 18 member cNNP. Note 16 of the structures were selected by the active learning and included in the training set of the cNNPs.

S4 On-The-Fly Training

S4.1 Downhill Monte Carlo Simulations

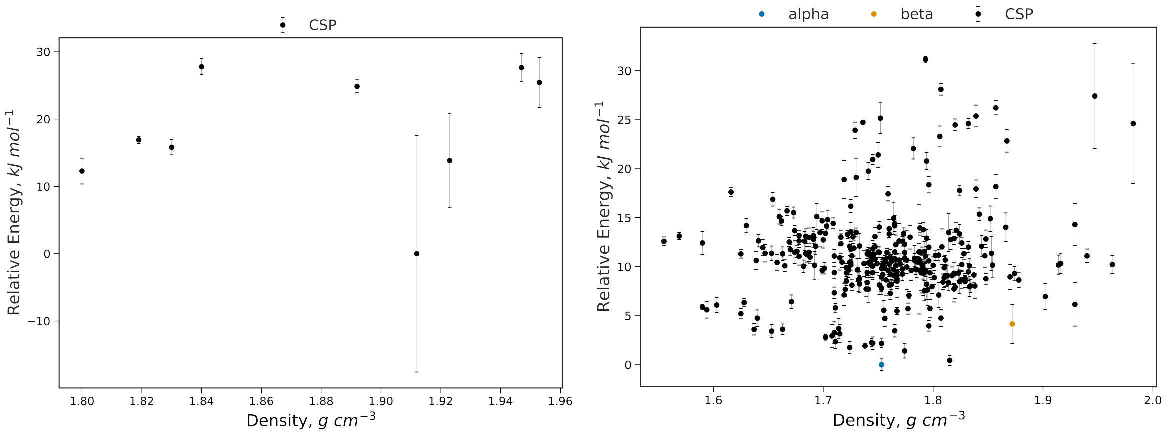

Figure S4: Oxalic acid landscape after downhill Monte Carlo using cNNPs trained on just the CSP minima (left) and with additional the Monte-Carlo on-the-fly training (right). With the cNNP trained on CSP minima only 9 trajectories remained stable after 1500 MC steps. By contrast only one trajectory became unstable with the on-the-fly trained cNNP.

S4.2 On-The-Fly Sampling including trajectory from the α polymorph of Oxalic Acid

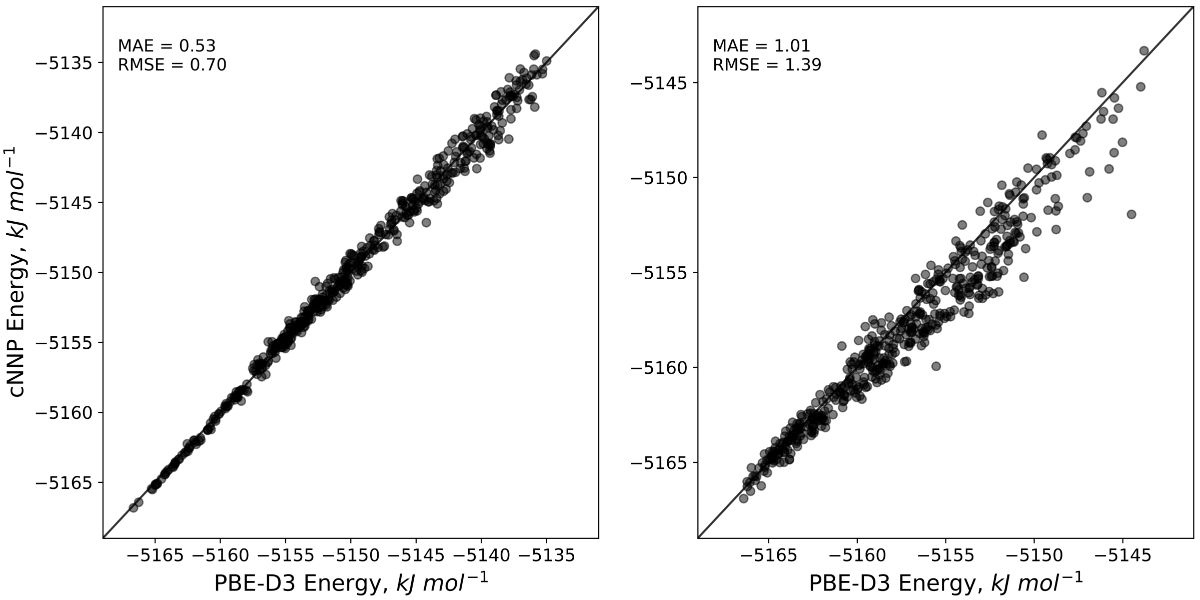

Figure S5: Correlation of cNNP and PBE-D3 for the α sampled (left) and β sampled (right) test structures following on the fly training including the α form.

S4.3 On-The-Fly Training with 10.0 kJ mol^{-1} Uncertainty Threshold

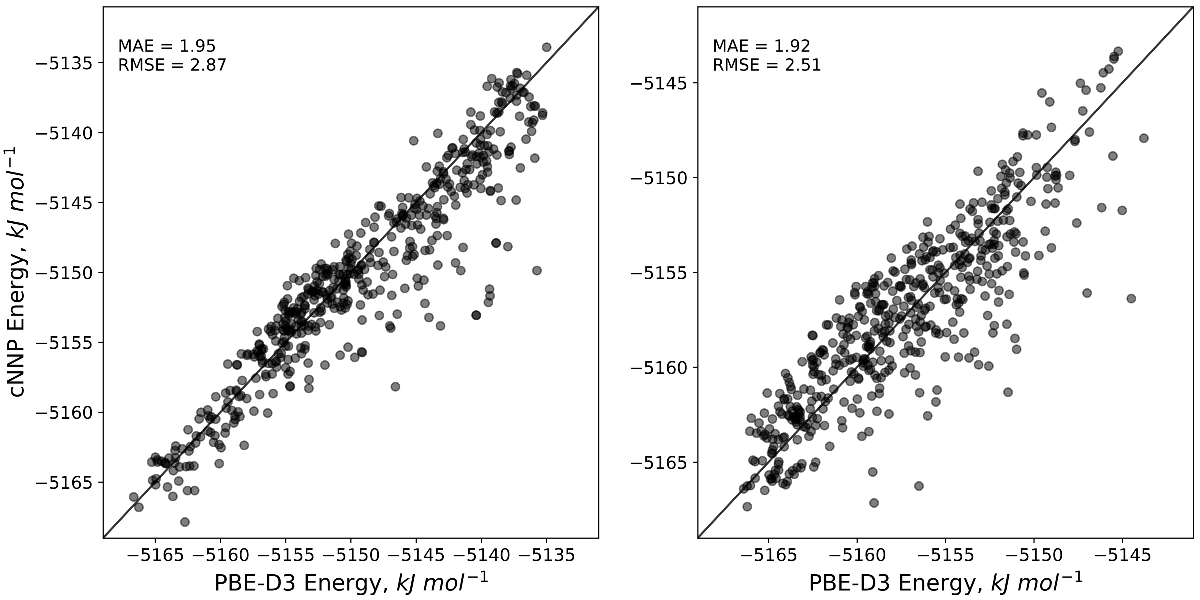

Figure S6: Correlation of cNNP and PBE-D3 for the α sampled (left) and β sampled (right) test structures following on-the-fly training from the 10 furthest point sampled structures with an uncertainty threshold of 10 kJ mol^{-1}.

References

(1) Case, D. H.; Campbell, J. E.; Bygrave, P. J.; Day, G. M. *J. Chem. Theory Comput.* **2016**, *12*, 910–924.

(2) Williams, D. E.; Cox, S. R. *Acta Cryst B* **1984**, *40*, 404–417.

(3) Frisch, M. J. et al. Gaussian09 Revision D.01, Gaussian Inc. Wallingford CT: Gaussian Inc., 2013.

(4) Stone, A. J. *J. Chem. Theory Comput.* **2005**, *1*, 1128–1132.

(5) Ferenczy, G. G. *J. Comput. Chem.* **1991**, *12*, 913–917.

(6) Holden, J. R.; Du, Z.; Ammon, H. L. *J. Comput. Chem.* **1993**, *14*, 422–437.

(7) Price, S. L.; Leslie, M.; Welch, G. W. A.; Habgood, M.; Price, L. S.; Karamertzanis, P. G.; Day, G. M. *Phys. Chem. Chem. Phys.* **2010**, *12*, 8478–8490.

(8) Hourahine, B. et al. *J. Chem. Phys.* **2020**, *152*, 124101.

(9) Brandenburg, J. G.; Grimme, S. *J. Phys. Chem. Lett.* **2014**, *5*, 1785–1789.

(10) Grimme, S.; Ehrlich, S.; Goerigk, L. *Journal of computational chemistry* **2011**, *32*, 1456–1465.

(11) Kresse, G.; Hafner, J. *Physical review B* **1993**, *47*, 558.

(12) Kresse, G.; Furthmüller, J. *Computational materials science* **1996**, *6*, 15–50.

(13) Kresse, G.; Furthmüller, J. *Physical review B* **1996**, *54*, 11169.

(14) Kresse, G.; Joubert, D. *Physical review b* **1999**, *59*, 1758.
